# Pregnancy Intentions and Maternal Health Behaviours: Observational Study in 18 African Countries

**DOI:** 10.1111/1471-0528.18367

**Published:** 2025-09-10

**Authors:** Bolanle Olapeju, Anna Maria van Eijk, Michael Bride, Anna Passaniti, Saade Ahmed Abdallah, Gabrielle C. Hunter, Safia Mohammed, Leila Kaze, Judith Nalukwago, Zoé Mistrale Hendrickson

**Affiliations:** ^1^ Department of Preventive Medicine and Biostatistics Uniformed Services University of the Health Sciences Bethesda Maryland USA; ^2^ Liverpool School of Tropical Medicine Liverpool UK; ^3^ Johns Hopkins Center for Communication Programs Baltimore Maryland USA; ^4^ PwC Kenya Nairobi Kenya; ^5^ Zanzibar Malaria Elimination Program Ministry of Health Zanzibar Tanzania; ^6^ Burundi National Malaria Control Program Bujumbura Burundi; ^7^ Makerere University Kampala Uganda; ^8^ Department of Behavioral and Community Health Sciences University of Pittsburgh School of Public Health Pittsburgh Pennsylvania USA

**Keywords:** Africa, behaviour, integration, intentions, pregnancy, prevention

## Abstract

**Objective:**

This study explores the relationship between pregnancy intentions and maternal health behaviours.

**Design and Setting:**

Secondary data analysis of recent (2018–2023), cross‐sectional demographic and health surveys from 18 sub‐Saharan African countries.

**Population:**

Survey respondents were women aged 15–49 years old with a child less than a year old who responded to survey questions about their pregnancy intentions for that child (*N* = 39 936).

**Methods:**

Logistic regressions and meta‐analysis using fixed effects explored the relationship between pregnancy intentions and maternal health behaviours across study countries adjusting for sociodemographic and contextual variables.

**Measures:**

Pregnancy intentions were defined as intended versus unintended. Maternal health behaviours included (i) early ANC, (ii) 4+ ANC contacts (ANC4+), (iii) 3+ doses of intermittent preventive treatment of malaria in pregnancy (IPTp3+), (iv) Mosquito net use in pregnancy, (v) receipt of tetanus toxoid and (vi) immediate breastfeeding.

**Results:**

Overall, 25% of women did not want their index pregnancy (ranging from 13% in Burkina Faso to 49% in Gabon). Unintended pregnancies were associated with reduced odds of early ANC (aOR: 0.68, 95% CI: 0.63, 0.73), ANC4+ (AOR: 0.70; 95% CI: 0.65, 0.75), IPTp3+ (AOR: 0.87; 95% CI: 0.81, 0.94), receiving tetanus toxoid (AOR: 0.74; 95% CI: 0.68, 0.80) and immediate breastfeeding (AOR: 0.83; 95% CI: 0.80, 0.87).

**Conclusions:**

Study findings corroborate the role of reproductive health agency and pregnancy preparedness in optimising maternal health behaviours and subsequent outcomes. Integration of reproductive health services, malaria service delivery and social and behaviour change interventions can help to improve pregnancy outcomes.

## 1 Introduction

### 1.1 Background

Unintended pregnancies and adverse maternal and neonatal health outcomes are global public health challenges that may be systematically associated with each other [1, 2, 3, 4]. The persistence of unintended pregnancies and adverse maternal, newborn and child health outcomes may point to systemic shortcomings in healthcare delivery systems [5]. Fragmented, siloed approaches to maternal, newborn and child health (MNCH) have historically separated reproductive health, malaria control, tuberculosis, HIV and MCH initiatives, undermining opportunities for integrated strategies [6, 7, 8]. This fragmentation is further reflected in funding streams and programmatic priorities that predominantly emphasise pregnancy and the postpartum period, with limited focus on pre‐pregnancy health promotion and prevention efforts [9, 10]. Reproductive health and MNCH are jointly impacted by social determinants and health service quality [7, 11, 12, 13, 14, 15, 16] across the continuum of care and life course [17, 18]. Thus, more attention should be placed on opportunities for synergy in intervention research, design, implementation, monitoring and evaluation.

### 1.2 Maternal Health Behaviours and Pregnancy Intentions

An underexplored correlate of maternal health behaviours is women's pregnancy intentions—defined as their desires and preferences regarding childbearing [19, 20, 21]. Unintended pregnancies may be mistimed (wanted later) or unwanted (not wanted at all). Women's desires regarding the timing and spacing of pregnancies can impact their health‐related behaviours during pregnancy. Isolated studies suggest that unintended pregnancies are linked with reduced uptake of recommended behaviours such as early [22, 23, 24, 25] and frequent antenatal care (ANC) contacts [22, 26], insecticide‐treated net (ITN) use [27], adherence to intermittent preventive treatment (IPTp) of malaria with sulfadoxine‐pyrimethamine (SP) [28, 29], postnatal care [18] and breastfeeding [30]. However, no study has systematically explored multiple maternal health behaviours that are associated with pregnancy intentions across sub‐Saharan Africa.

### 1.3 Study Objectives and Rationale

This study seeks to address critical knowledge gaps by exploring the relationship between pregnancy intentions and several maternal health behaviours (early and frequent ANC, receipt of tetanus toxoid or IPTp, mosquito net use during pregnancy and immediate breastfeeding after delivery) in sub‐Saharan Africa. Theoretical underpinnings are drawn from self‐determination theory [31] which posits that intentions act as a motivational determinant of behaviour. A woman may be more likely to actively adopt maternal health behaviours when she feels she has control over her reproductive choices (autonomy), believes she is capable of engaging in maternal health behaviours (competence) and feels supported by significant others (e.g., spouse, family or friends) in her decision (relatedness). Thus, having an intended pregnancy can serve to motivate women to engage in health behaviours that ensure that she and her baby remain healthy. In addition, women with unintended pregnancies may have other presumable constraints, such as not planning their family, not utilising relevant reproductive health services and not using contraceptive methods effectively among others. They may also have less coping and agency capability, making them altogether more vulnerable. We hypothesised that women who did not intend to be pregnant would be less likely to engage in specific behaviours to prevent adverse maternal health outcomes, such as ANC, IPTp, mosquito net use and breastfeeding.

## 2 Methods

### 2.1 Design and Setting

This study was a cross‐sectional study design and used secondary data analysis of recent (2018–2023) Demographic and Health Surveys (DHS) in 18 sub‐Saharan African countries. Of note, study countries account for the majority (over 90%) of the global malaria burden [4]. DHS data are widely regarded as high quality and reliable due to their standardised methodology, rigorous sampling design and consistent implementation across countries and time periods [32]. The surveys use nationally representative samples and validated instruments to ensure comparability and accuracy, making them a trusted source for monitoring health and population trends in low‐ and middle‐income countries [33]. More information on DHS surveys, including official survey reports, publicly available data and standard questionnaires, can be found on the DHS program website—https://dhsprogram.com/.

### 2.2 Study Population

The DHS respondents include women of reproductive age (15–49). For this study, the sample included female survey respondents (i) aged 15–49 years old; (ii) with a child less than a year old; and (iii) who responded to survey questions about their pregnancy intentions for that child (*N* = 39 936). To specifically assess the relationship between pregnancy intentions and mosquito net use during pregnancy, the analysis included women (i) 15–49 years old with nets in their household; (ii) who were currently pregnant as of the time of the survey; and (iii) who responded to survey questions about their intentions for their current pregnancy (*N* = 6406).

### 2.3 Measures

The primary exposure variable was pregnancy intentions based on two survey questions for the index child: ‘When you got pregnant, did you want to get pregnant at that time?’ and ‘Did you want to have a baby later on or did you not want any (more) children?’. Responses were combined, and pregnancy intentions were defined as (i) intended, (ii) mistimed (i.e., wanted the child later); or (iii) unwanted (i.e., not wanted at all). This was subsequently dichotomized into intended versus unintended (mistimed or not wanted at all).

Maternal health behaviours included (i) early ANC defined as the first ANC visit within the first trimester, (ii) ANC retention (at least four contacts (ANC4+), (iii) receipt of three or more doses of SP (IPTp3+), (iv) receipt of one or more doses of tetanus toxoid immunisation, v) mosquito net use the night preceding the survey, and (vi) immediate breastfeeding (within the first hour of birth).

Sociodemographic and contextual factors were based on available DHS data and a priori knowledge of factors influencing pregnancy intentions and maternal health behaviours. Factors included age group (15–24, 25–34, and 35+ years), residence (urban vs. rural), religion (Christian vs. other), education level (secondary vs. less), marital status (married vs. not), sex of the head of household (male vs. female), wealth quintile (first [lowest] to fifth [highest]), number of children under 5 in the household, country, year of survey and location of last delivery (home vs. other). The DHS women's questionnaire detailing how all the variables were asked in the survey is included as Data S1.

### 2.4 Analysis

Data management and analysis was conducted using Stata version 17 (Stata Corporation, College Station, TX, USA). The *svyset* command in STATA was used to weight the data and ensure representation of the study population for each country by the cluster level. Crude and multivariable logistic regression models explored the relationship between pregnancy intentions and maternal health behaviours across study countries adjusting for the aforementioned contextual variables. A two‐stage meta‐analysis using fixed effects was conducted to pool the country estimates using the *metan* command. Heterogeneity in effect measures between countries was evaluated using the *I* [2] statistic. For two specific analyses (mosquito net use among pregnant women and immediate breastfeeding), the sample was too limited to allow adjustment for clustering, and analyses of unclustered data was conducted instead.

## 3 Results

### 3.1 Description of Study Sample

Table 1 summarises the characteristics of the study sample (*N* = 39 936), highlighting country‐level differences. Overall, about a third of the women lived in urban areas (34%), were aged 15–24 years (37%) and had a secondary education (34%). The majority of women were married (87%) while half of the women were Christian (49%). About a fifth (19%) of women lived in female‐headed households.

**TABLE 1:** Description of study sample.

Country	Sample		Sociodemographic characteristics (%)					
	Survey	*N*	Urban	15–24 years old	Secondary Education	Christian	Female HH	Married
Burkina Faso	2021 DHS	2366	29	37	20	31	8	96
Cameroon	2018 DHS	1902	44	42	47	67	20	80
Côte d'Ivoire	2021 DHS	2149	41	38	18	45	15	84
Gabon	2019–21 DHS	1285	69	41	73	83	37	62
Gambia	2019–20 DHS	1798	44	33	30	0	13	95
Ghana	2022 DHS	1891	39	32	56	69	27	86
Guinea	2018 DHS	1534	28	36	13	10	12	95
Kenya	2022 DHS	3958	34	40	48	86	28	82
Liberia	2019–20 DHS	1130	36	44	32	84	31	68
Madagascar	2021 DHS	2532	19	48	34	63	18	79
Mali	2018 DHS	1933	26	40	18	3	13	96
Mauritania	2019–21 DHS	2312	39	32	18	N/A	35	94
Nigeria	2018 DHS	6280	34	32	41	36	8	96
Rwanda	2019–20 DHS	1546	20	24	29	97	22	83
Senegal	2019 DHS	1255	29	34	20	0	22	96
Sierra Leone	2019 DHS	1931	31	38	33	20	23	85
Tanzania	2022 DHS	2174	27	38	30	N/A	22	85
Zambia	2018 DHS	1960	29	47	40	99	21	75
Total		39 936	34	37	34	49	19	87

Abbreviations: %: percentage; DHS, demographic and health survey; HH, head of household; N, number; N/A, not available.

### 3.2 Rates of Pregnancy Intentions and Maternal Health Behaviours Across Study Countries

Table 2 displays the overall and country‐level uptake of pregnancy intentions and maternal health behaviours including early ANC, ANC4+, receipt of tetanus toxoid, IPTp3+, mosquito net use and immediate breastfeeding. Considerable variation in intended, mistimed and unwanted pregnancies was seen across study countries. For example, in Burkina Faso, 87% of pregnancies were wanted, 11% were mistimed and 2% were not wanted at all. In contrast, Gabon shows lower wanted pregnancies at 51%, with 36% mistimed and 31% unwanted.

**TABLE 2:** Prevalence of pregnancy intentions and maternal health behaviours.

Country	Pregnancy intentions (%)			Maternal, newborn and child health behaviours (%)					
	Intended	Mistimed	Unwanted	Early ANC	ANC4+	IPTp3+	Mosquito net use in pregnancy	Tetanus toxoid	Immediate breastfeeding
Burkina Faso	87	11	2	51	72	55	74	85	61
Cameroon	77	19	4	38	62	30	68	74	49
Côte d'Ivoire	73	23	4	37	56	34	N/A	85	45
Gabon	51	36	13	65	77	36	30	81	74
Gambia	78	19	3	42	79	54	48	84	34
Ghana	60	31	9	61	87	63	55	86	51
Guinea	82	15	4	27	35	37	31	79	39
Kenya	59	33	8	28	66	6	N/A	88	64
Liberia	55	37	8	70	86	40	49	92	67
Madagascar	86	9	5	28	56	33	63	67	61
Mali	82	15	3	37	44	28	90	75	67
Mauritania	78	17	6	57	38	11	17	61	58
Nigeria	88	10	3	18	56	16	63	71	43
Rwanda	57	30	13	56	48	N/A	66	72	86
Senegal	84	14	2	60	54	21	77	88	23
Sierra Leone	80	17	3	44	79	33	69	98	77
Tanzania	69	27	3	33	64	33	71	79	69
Zambia	56	37	7	36	61	59	53	75	78
Total	75	20	5	39	61	30	61	78	57

Abbreviations: %, percentage; ANC, antenatal care; DHS: demographic and health survey; IPTp3+: intermittent preventive treatment of malaria in pregnancy; N/A: not available.

Overall, 39% of women in the study population attended their first ANC visit within the first trimester, ranging from 18% in Nigeria to 70% in Liberia. Over three‐quarters (78%) of women received tetanus toxoid immunisation during their pregnancy, which was lowest in Mauritania (61%) and highest in Sierra Leone (98%). In contrast, less than a third (30%) of women had IPTp3+, ranging from 11% in Nigeria to 63% in Ghana. More than half (57%) of women breastfed their babies immediately (within the first hour of birth), which was lowest in Senegal (23%) and highest in Rwanda (86%).

Sixty‐one per cent of women had at least four ANC contacts, which was lowest in Guinea (35%) and highest in Ghana (87%). Similarly, 61% of currently pregnant women used a mosquito net the night before the survey, which was lowest in Mauritania (22%) and highest in Mali (88%).

### 3.3 Relationship Between Pregnancy Intentions and Maternal Health Behaviours Across Study Countries

**FIGURE 1:**
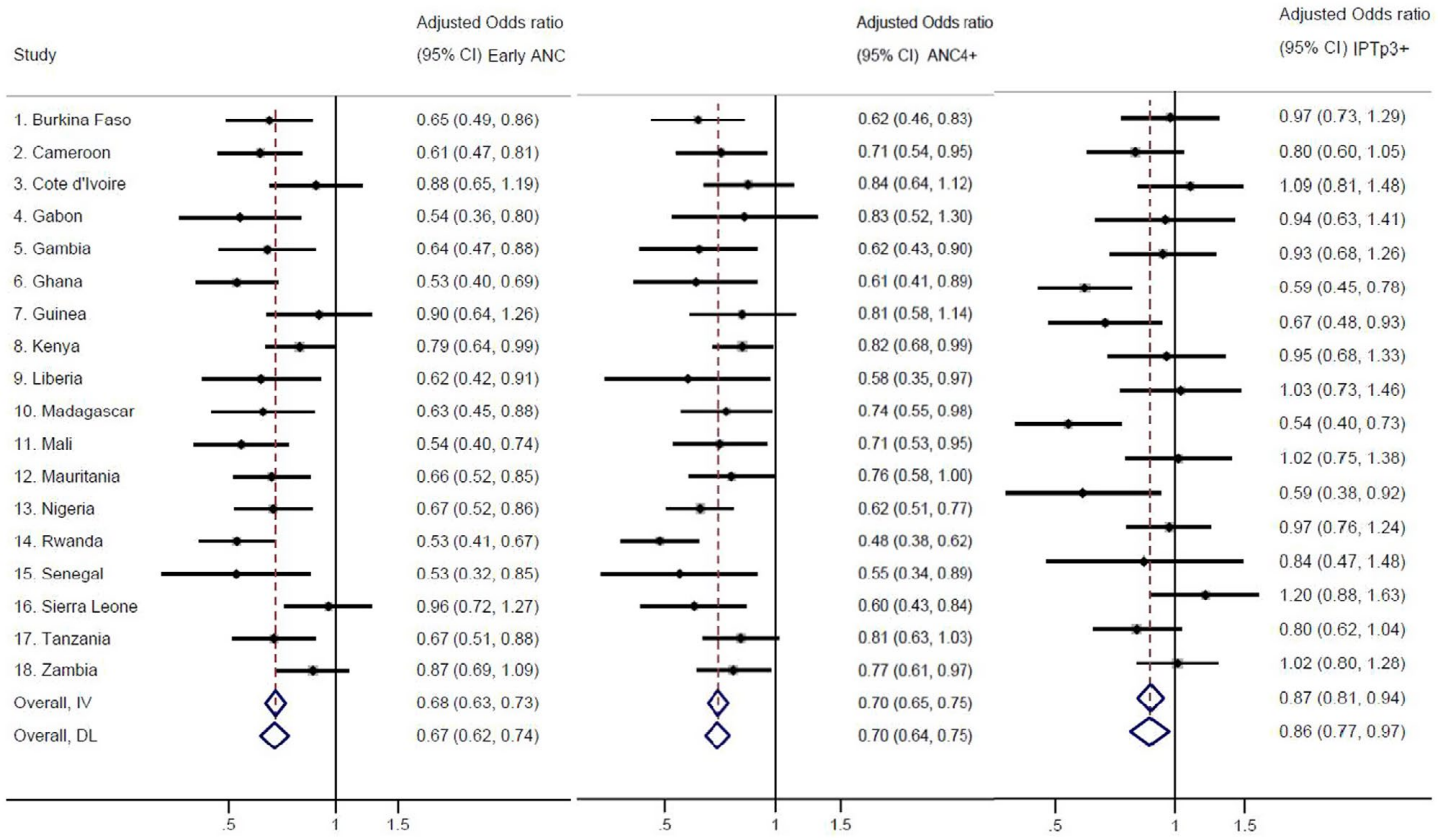
Pregnancy intentions and early ANC, ANC4+ and IPTp3+. IV stands for fixed effects meta‐analysis and DL for random effects model. Complete information in the supplement, Figures S2–, S4. Interpretation example: Compared to women with a planned pregnancy, women with an unintended pregnancy in Burkina Faso were less likely to attend the ANC early (adjusted odds ratio [aOR] 0.65, 95% CI 0.49–0.86), were less likely to attend the ANC 4 or more time (aOR 0.62, 0.46–0.83) but had a similar chance to receive IPTp3 (aOR 0.97, 0.73–1.29).

Multivariable regression analyses explored the relationships between pregnancy intentions and maternal health behaviours, adjusting for the following sociodemographic and contextual covariates: age, residence, religion, education, marital status, sex of the head of household, wealth, number of children under five in the household, country, year of survey and location of last birth. Figure 1 displays forest plots that summarise the relationship between pregnancy intention and early ANC, ANC4+ visits and IPTp3+ respectively, across study countries (See Figures [Link], [Link] for more detailed results). Women whose recent pregnancies were unintended had 32% lower odds of early ANC (AOR: 0.68; 95% CI: 0.63, 0.73) overall, as compared to those who reported intended pregnancies. At the country level, this significant association was seen in most countries except for Côte d'Ivoire, Guinea, Sierra Leone and Zambia. Similarly, women with unintended pregnancies had 30% less odds of ANC4+ (AOR: 0.70; 95% CI: 0.65, 0.75) overall; however, this association was not significant in Côte d'Ivoire, Gabon, Guinea and Tanzania. Women with unintended pregnancies had 13% lower odds of IPTp3+ (AOR: 0.87; 95% CI: 0.81, 0.94) overall, although the odds were only statistically significant in four countries. These included Ghana, Guinea, Madagascar and Mauritania.

**FIGURE 2:**
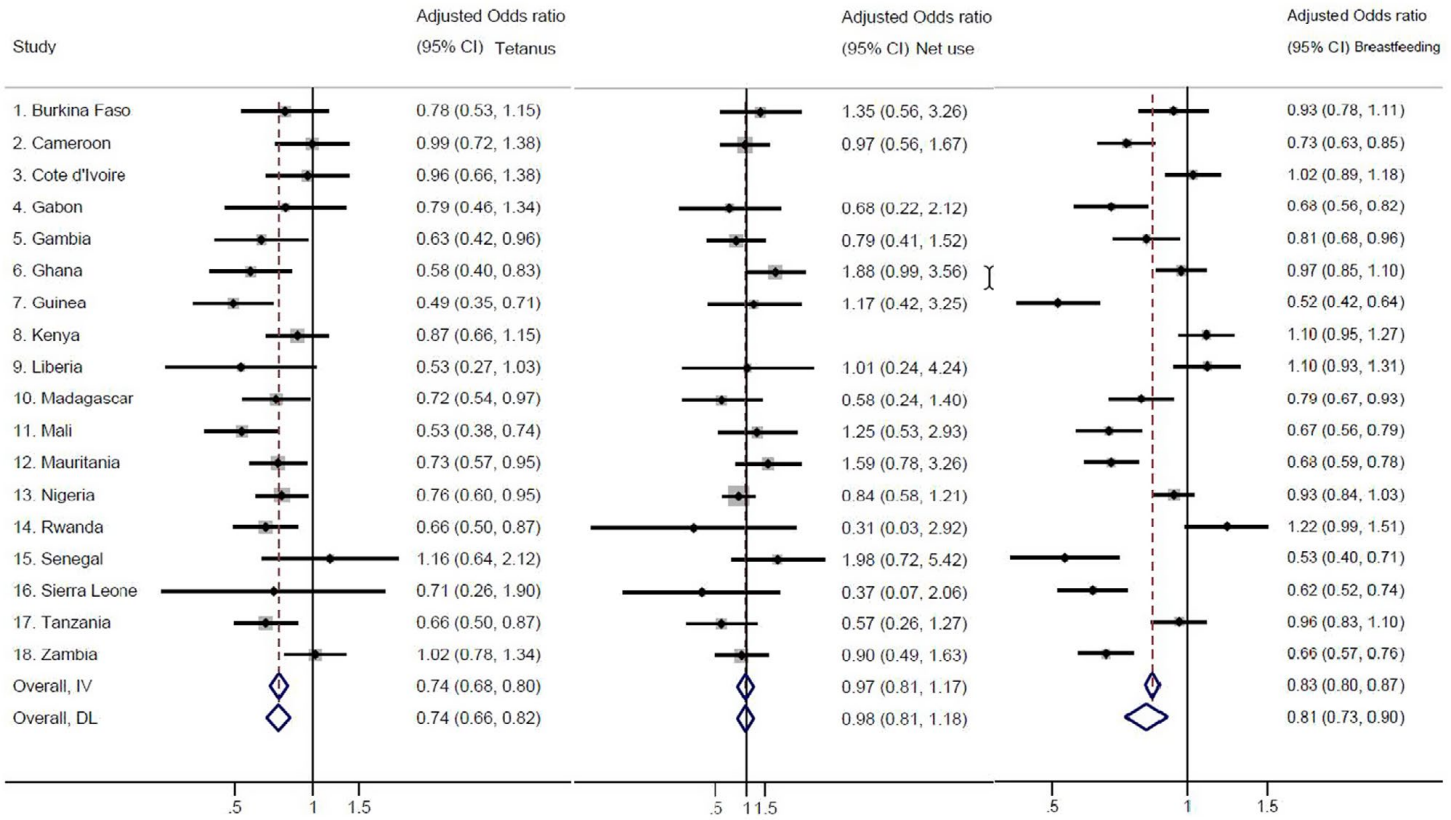
Pregnancy intentions and receipt of antenatal tetanus toxoid, mosquito net use in pregnancy and immediate breastfeeding. IV stands for fixed effects meta‐analysis and DL for random effects model. Complete information in the supplement Figures [Link], [Link]. Interpretation example: Compared to women with a planned pregnancy, women with an unintended pregnancy in Burkina Faso were less likely (but not significantly) to receive tetanus injections (aOR 0.78, 95% CI 0.53–1.15), were more likely (but not significantly) to sleep under a net (aOR 1.35, 0.56–3.26) and had a similar chance to start breastfeeding early (aOR 0.93, 0.78–1.11).

Figure 2 displays forest plots that summarise the relationship between pregnancy intention and tetanus injections, net use in pregnancy and immediate breastfeeding, respectively, across study countries (See Figures [Link], [Link] for more detailed results). Women whose recent pregnancies were unintended had 24% lower odds of receiving tetanus toxoid (AOR: 0.74; 95% CI: 0.68, 0.80) overall. This association was, however, not significant in several countries: Burkina Faso, Cameroon, Côte d'Ivoire, Gabon, Kenya, Liberia, Senegal, Sierra Leone and Zambia. Having an unintended recent pregnancy was not significantly associated with mosquito net during pregnancy (AOR: 0.97; 95% CI: 0.81, 1.17). Finally, women with unintended recent pregnancies were less likely to immediately breastfeed (AOR: 0.83; 95% CI: 0.80, 0.87) overall, and this was also not significant in several countries: Burkina Faso, Côte d'Ivoire, Ghana, Kenya, Liberia, Nigeria, Rwanda and Tanzania.

## 4 Discussion

### 4.1 Main Findings

This study explored the relationship between pregnancy intentions and several maternal health behaviours across multiple countries in sub‐Saharan Africa. While there was notable country‐level heterogeneity, women with unintended pregnancies were significantly less likely to access early or frequent antenatal care, receive tetanus toxoid or get intermittent preventive malaria treatment. Of note, the study did not see an association between pregnancy intentions and mosquito net use, presumably due to limitations in the DHS methodology to assess ITN use [34] among other unknown reasons.

### 4.2 Interpretations

Global data suggest that differences in unintended pregnancies may be influenced by a complex interplay of socioeconomic, cultural and healthcare‐related factors such as poverty and stalled economic development, harmful norms related to fertility or use of reproductive health services, lack of sexual reproductive health care and information, sexual violence and reproductive coercion [35]. Similarly, global variations in the study outcomes (Early ANC, ANC4+, mosquito net use, receipt of tetanus toxoid and IPTp3+ and immediate breastfeeding) have been explained in part by multilevel factors at the individual and household [36], community [37, 38] and health system service delivery [39, 40]. Study findings also complement research conducted in other settings such as the United States where unwanted births were less likely to receive early antenatal care or be breast‐fed, and more likely to be low birth weight [41, 42].

While this study assesses associations and not causal relationships between pregnancy intentions and MNCH behaviours, there are plausible pathways linking pregnancy intention and MNCH behaviours. A woman who is unintentionally pregnant may not recognise her pregnancy early [43]. In addition, she may not want her pregnancy status to be known and may hold back from receiving health services as her status would be disclosed and become publicly known. Furthermore, unintended pregnancies might be associated with younger age, lack of economic resources or social support [11], and this might impact a woman's ability to access MNCH care services. These pathways are somewhat reflected in the self‐determination theory [31] as a woman's control over her reproductive choices (autonomy) is linked to her ability (competence) to engage in MNCH behaviours (competence), and her support (relatedness) from significant others (e.g., spouse, family or friends). In addition to the Self‐Determination Theory, theories like the Theory of Planned Behavior [44] and the Health Belief Model [45] can provide frameworks for understanding how intentions, beliefs, social influences and perceived control can shape pregnant women's health behaviours. Other pathways explicitly explored in other settings include the Compensatory Carry‐Over Action Model [46] which connects health behaviours with intention, planning, self‐efficacy, social support and discrimination.

The association observed between pregnancy intentions and maternal behaviours underscores the relevance of integrating reproductive health considerations into malaria prevention and broader MNCH strategies. Study findings suggest that interventions focused on the pre‐pregnancy period and women's pregnancy intentions may serve as an entry point for improving maternal and child health. Previous studies have demonstrated that culturally sensitive family planning programmes, including those that take a gender transformative approach [47] or centre women's reproductive health agency, can help women to meet their reproductive goals and reduce unintended pregnancy [48, 49, 50]. The potential gains in preventing malaria could be yet another benefit to improved reproductive health agency, along with other health and educational benefits for women and their families.

### 4.3 Implications

The results of this study highlight the potential utility of cross‐sectoral program models for advancing MNCH and malaria elimination in sub‐Saharan Africa. Existing vertical health programmes may benefit from more integrated planning approaches that include reproductive health, malaria, HIV and adolescent health services. Donor alignment and coordinated funding strategies could support such integration by facilitating joint programming across sectors, avoiding implementation of siloed malaria interventions separately from reproductive, maternal, newborn, child and adolescent health and HIV [51]. For example, multilateral bodies such as the African Union have called for collaboration among key development partners, including the World Bank, Global Fund, United Nations Children's Fund (UNICEF) and United Nations Population Fund (UNFPA) to promote multisectoral investments that span gender, youth and health domains [17].

All the MNCH behaviours explored in this study were directly linked to health service delivery, suggesting a need to examine the organisational structure and programmatic coordination of MNCH, malaria and reproductive health services at national and subnational levels. In contexts where these services are managed under separate administrative units, a unified strategic framework may enhance collaboration, coherence and efficiency [52]. Institutional mechanisms such as interdepartmental technical working groups may facilitate joint planning, implementation and monitoring [53]. Joint data measurement and action reviews may help identify and track priority indicators for malaria, MNCH and reproductive health [54]. Additionally, harmonised measurement and joint data reviews may support improved tracking of priority indicators and promote coordinated program responses to address identified service gaps.

Tailored, context‐specific programmatic approaches may also support improved integration of reproductive health into malaria and MNCH platforms. Community‐focused approaches [55] that are informed by life course theory [56] and stratify pregnancy preparedness interventions for younger women versus older women [57] may be particularly relevant. For instance, interventions targeting adolescents and young adults may prioritise preconception health and pregnancy preparedness, while those targeting older women may focus on interpregnancy intervals and safe pregnancy planning [49, 58]. Some evidence suggests that conditional cash transfers and youth‐friendly health services may increase contraceptive use and reduce unintended pregnancies among adolescents in low‐ and middle‐income settings [58]. Integration of preconception counselling into school‐based or adolescent health services may also be beneficial [50]. Similarly, community‐level demand‐side interventions, including health promotion activities such as ‘wellness days’ [47], may be useful for improving uptake of both reproductive and malaria‐related services among broader community populations.

The relatively low prevalence of MNCH behaviours—excluding tetanus toxoid coverage—across countries included in this analysis points to the continued need for demand generation and supply‐side improvements. Study findings corroborate the need for targeted interventions to address barriers, raise awareness and strengthen healthcare accessibility [59], especially in settings where early ANC attendance remains low, given that early ANC attendance is a strong predictor for ANC4 and, relatedly, IPTp3. In addition, pregnant women are given insecticide‐treated nets during ANC in several countries. Demand generation interventions and social and behaviour change strategies may help position intended pregnancies as helping to prevent malaria and ensure the health of mothers and babies [60]. Social and behaviour change interventions may also address stigma surrounding unintended pregnancy, which may lead women, particularly young women, to keep their pregnancies a secret and be a barrier to care‐seeking [61]. These demand‐side approaches should be complemented with improvements in service delivery, including the provision of respectful, high‐quality care and consistent commodity availability [60]. Lessons from tetanus toxoid immunisation programmes, which achieved relatively high coverage across most settings, may offer useful implementation insights for scaling similar success in IPTp and ITN interventions [62].

Study findings also suggest directions for future research. Longitudinal or advanced modelling studies may be conducted to better quantify the value of integrating reproductive health into malaria and MNCH using measures such as lives saved or malaria cases averted from planned pregnancies. Where possible, existing health management information systems can be employed to identify and track relevant available data related to malaria in pregnancy and MNCH. Countries should investigate opportunities for intra‐ and international research collaboration efforts to understand past and current efforts to integrate malaria, MNCH and reproductive health to better understand what works well or does not work well.

### 4.4 Strengths and Limitations

The study boasts some strengths. These include the scope of the study population, which included women from 18 sub‐Saharan African countries; the use of well‐validated and standardised data from the DHS, which employs population‐based, nationally representative samples that also allow for cross‐country comparisons; and the focus on maternal and child health behaviours, including reproductive health, malaria prevention and nutrition behaviours. However, there are also a number of limitations to be acknowledged that should be addressed by more robust studies. This snapshot of national‐level data does not fully explore inter‐country differences or country profiles and does not take into account heterogeneity in sub‐national health policies related to the outcomes of interest. Future analyses could build on the findings presented here to explore similarities and differences in country profiles in greater depth. For example, rates of IPTp3+ and ITN use are highly sensitive to access to these interventions. Most countries distribute ITNs only every 3 years, and drug stock shortages occur commonly [63]. An analysis of these contextual differences would be an important step to inform future intervention design or policy decisions. Furthermore, the cross‐sectional nature of this study does not allow inferences of causality, and we are careful to position our findings as preliminary. Only variables available in the DHS were used in this analysis, limiting the study's ability to fully explore key constructs in self‐determination theory. Pregnancy intention was used as a proxy for a woman's autonomy in her pregnancy decisions while engaging in related MNCH behaviour was a proxy for her competence to take action. However, social support was not well explored, and pregnancy intentions are not necessarily representative of all domains of autonomy. The study focuses on the adoption of relevant behaviours that might be influenced by the access to and quality of health services but does not explore population‐level health outcomes such as mortality. Lastly, all data are self‐reported and thus subject to social desirability bias, particularly in settings where it is not acceptable to say that pregnancies are unwanted or mistimed.

## 5 Conclusion

This study across 18 countries in sub‐Saharan Africa demonstrated that women who intended to be pregnant were more likely to have beneficial maternal health behaviours. Study findings suggest the importance of empowering women to optimise their reproductive health and engage in behaviours that ensure healthy mothers and babies. In addition, the integration of reproductive health services, malaria service delivery and behaviour change interventions can help to improve pregnancy intentions and outcomes.

## Author Contributions

B.O. conceived the study. B.O., A.V.E. and M.B. managed the datasets and analysed the data. B.O., A.V.E., M.B., A.P., S.A.A., G.H., S.M., L.K., J.N. and Z.M.H. drafted, edited and approved the final manuscript.

## Ethics Statement

The authors have nothing to report.

## Consent

The authors have nothing to report.

## Conflicts of Interest

The authors declare no conflicts of interest.

## Supporting information


**Data S1:** DHS questionnaire.

**Figure S2:** Pregnancy intentions and early ANC.

**Figure S3:** Pregnancy intentions and ANC4+.

**Figure S4:** Pregnancy intentions and IPTp3+.

**Figure S5:** Pregnancy intentions and receipt of tetanus toxoid.

**Figure S6:** Pregnancy intentions and mosquito net use during pregnancy.

**Figure S7:** Pregnancy intentions and immediate breastfeeding.

## Data Availability

The datasets are publicly available from the DHS Program web site, http://www.dhsprogram.com.
