# Supplementary material for: Pregnancy Intentions and Maternal Health Behaviours: Observational Study in 18 African Countries
Source: BJOG. 2025 Sep 10;132(13):2246–55. doi: 10.1111/1471-0528.18367 (PMC12592751; doi:10.1111/1471-0528.18367)
Supplement: Supplementary file 1 — Data S1: DHS questionnaire. [file BJO-132-2246-s001.docx]

[NAME OF COUNTRY] [NAME OF ORGANIZATION]

|  |  |  |  |
| --- | --- | --- | --- |
|  |  |  |  |
|  | |  |  |

DEMOGRAPHIC AND HEALTH SURVEYS MODEL WOMAN'S QUESTIONNAIRE

FORMATTING DATE: ENGLISH LANGUAGE:

17 Dec 2018

17 Dec 2018

| **IDENTIFICATION (1)** | | | | | | |
| --- | --- | --- | --- | --- | --- | --- |
| PLACE NAME NAME OF HOUSEHOLD HEAD CLUSTER NUMBER . . . . . . . . . . . . . . . . . . . . . . . . . . . . . . . . . . . . . . . . . . . . . . . . . . . . . . . . . . . . . . . . . . . . . . . .  HOUSEHOLD NUMBER . . . . . . . . . . . . . . . . . . . . . . . . . . . . . . . . . . . . . . . . . . . . . . . . . . . . . . . . . . . . . . . . . . .  NAME AND LINE NUMBER OF WOMAN | | | | | | |
| **INTERVIEWER VISITS** | | | | | | |
|  | 1 | 2 | 3 | FINAL VISIT | | |
| DATE  INTERVIEWER'S NAME  RESULT* |  |  |  | DAY MONTH YEAR INT. NO. RESULT* | | |
| NEXT VISIT: DATE  TIME |  |  |  | TOTAL NUMBER OF VISITS | | |
| *RESULT CODES: 1 COMPLETED 4 REFUSED   1. NOT AT HOME 5 PARTLY COMPLETED 7 OTHER 2. POSTPONED 6 INCAPACITATED SPECIFY | | | | | | |
| LANGUAGE OF **0 1** LANGUAGE OF NATIVE LANGUAGE TRANSLATOR USED QUESTIONNAIRE** INTERVIEW** OF RESPONDENT** (YES = 1, NO = 2)  LANGUAGE OF  **ENGLISH** **LANGUAGE CODES:  QUESTIONNAIRE** 01 ENGLISH 03 LANGUAGE 3 05 LANGUAGE 5  02 LANGUAGE 2 04 LANGUAGE 4 06 LANGUAGE 6 | | | | | | |
| SUPERVISOR  NAME NUMBER | | FIELD EDITOR  NAME NUMBER | | | OFFICE EDITOR  NUMBER | KEYED BY  NUMBER |

(1) This section should be adapted for country-specific survey design.

|  |  |  |  |
| --- | --- | --- | --- |

|  |  |  |  |
| --- | --- | --- | --- |

Note: Questions with blue highlighting in the question number column are HIV-related questions that may be deleted in some circumstances (see footnotes). Questions with pink highlighting in the question number column are malaria-related questions that may be deleted in some circumstances (see footnotes). Questions with yellow highlighting in the question number column are other questions that may be deleted in some circumstances (see footnotes). Brackets [ ] indicate items that should be adapted on a country-specific basis.

INTRODUCTION AND CONSENT

|  |  |  |  |
| --- | --- | --- | --- |

(1)

Hello. My name is . I am working with [NAME OF ORGANIZATION]. We are conducting a survey about health and other topics all over [NAME OF COUNTRY]. The information we collect will help the government to plan health services. Your household was selected for the survey. The questions usually take about 30 to 60 minutes. All of the answers you give will be confidential and will not be shared with anyone other than members of our survey team. You don't have to be in the survey, but we hope you will agree to answer the questions since your views are important. If I ask you any question you don't want to answer, just let me know and I will go on to the next question or you can stop the interview at any time.

In case you need more information about the survey, you may contact the person listed on the card that has already been given to your household.

Do you have any questions? May I begin the interview now?

SIGNATURE OF INTERVIEWER DATE

| RESPONDENT AGREES |  | RESPONDENT DOES NOT AGREE |  |
| --- | --- | --- | --- |
| TO BE INTERVIEWED . . | 1 | TO BE INTERVIEWED . . 2 | END |

SECTION 1. RESPONDENT'S BACKGROUND

|  |  |
| --- | --- |
|  |  |

| NO. | QUESTIONS AND FILTERS | CODING CATEGORIES | SKIP |
| --- | --- | --- | --- |
| 101 | RECORD THE TIME. | HOURS . . . . . . . . . . . . . . . . . . . . . . . .  MINUTES . . . . . . . . . . . . . . . . . . . . . . . . |  |
| 102 | How long have you been living continuously in (NAME OF CURRENT CITY, TOWN OR VILLAGE OF RESIDENCE)?  IF LESS THAN ONE YEAR, RECORD ‘00’ YEARS. | YEARS . . . . . . . . . . . . . . . . . . . . . . . .  ALWAYS 95  VISITOR 96 | 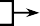 105 |
| 103 | Just before you moved here, did you live in a city, in a town, or in a rural area? | CITY 1  TOWN 2  RURAL AREA 3 |  |
| 104 | Before you moved here, which [PROVINCE/REGION/STATE] did you live in? | [PROVINCE/REGION/STATE] 01  [PROVINCE/REGION/STATE] 02  [PROVINCE/REGION/STATE] 03  OUTSIDE OF [COUNTRY] 96 |  |
| 105 | In what month and year were you born? | MONTH . . . . . . . . . . . . . . . . . . . . . . . .  DON'T KNOW MONTH 98  YEAR . . . . . . . . . . . . . . . .  DON'T KNOW YEAR 9. 998 |  |
| 106 | How old were you at your last birthday?  COMPARE AND CORRECT 105 AND/OR 106 IF INCONSISTENT. | AGE IN COMPLETED YEARS . . . . . . . . |  |
| 107 | Have you ever attended school? | YES 1  NO 2 | 111 |
| 108  (2) | What is the highest level of school you attended: primary, secondary, or higher? | PRIMARY 1  SECONDARY 2  HIGHER 3 |  |

| NO. | QUESTIONS AND FILTERS | | | CODING CATEGORIES | | SKIP |
| --- | --- | --- | --- | --- | --- | --- |
| 109  (2) | What is the highest [GRADE/FORM/YEAR] you completed at that level?  IF COMPLETED LESS THAN ONE YEAR AT THAT LEVEL, RECORD '00'. | | | [GRADE/FORM/YEAR] . . . . . . . . . . . . . | |  |
| 110 | CHECK 108: | PRIMARY OR SECONDARY 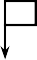 | HIGHER 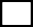 | |  | 113 |
| 111  (3) | Now I would like you to read this sentence to me. SHOW CARD TO RESPONDENT.  IF RESPONDENT CANNOT READ WHOLE SENTENCE,  PROBE: Can you read any part of the sentence to me? | | | CANNOT READ AT ALL . . . . . . . . . . . . . . . . . . . ABLE TO READ ONLY PART OF  THE SENTENCE. . . . . . . . . . . . . . . . . . . . . . . . ABLE TO READ WHOLE SENTENCE . . . . . . . . . . NO CARD WITH REQUIRED  LANGUAGE  (SPECIFY LANGUAGE) BLIND/VISUALLY IMPAIRED . . . . . . . . . . . . . . . . | 1  2  3  4  5 |  |
| 112 | CHECK 111: | CODE '2', '3'  OR '4' CIRCLED 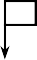 | CODE '1' OR '5'  CIRCLED 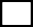 | |  | 114 |
| 113 | Do you read a newspaper or magazine at least once a week, less than once a week or not at all? | | | AT LEAST ONCE A WEEK . . . . . . . . . . . . . . . . . . .  LESS THAN ONCE A WEEK . . . . . . . . . . . . . . . . NOT AT ALL . . . . . . . . . . . . . . . . . . . . . . . . . . . | 1  2  3 |  |
| 114 | Do you listen to the radio at least once a week, less than once a week or not at all? | | | AT LEAST ONCE A WEEK . . . . . . . . . . . . . . . . . . .  LESS THAN ONCE A WEEK . . . . . . . . . . . . . . . . NOT AT ALL . . . . . . . . . . . . . . . . . . . . . . . . . . . | 1  2  3 |  |
| 115 | Do you watch television at least once a week, less than once a week or not at all? | | | AT LEAST ONCE A WEEK . . . . . . . . . . . . . . . . . . .  LESS THAN ONCE A WEEK . . . . . . . . . . . . . . . . NOT AT ALL . . . . . . . . . . . . . . . . . . . . . . . . . . . | 1  2  3 |  |
| 116 | Do you own a mobile telephone? | | | YES . . . . . . . . . . . . . . . . . . . . . . . . . . . . . . . . . . . .  NO . . . . . . . . . . . . . . . . . . . . . . . . . . . . . . . . . . . . | 1  2 | 118 |
| 117 | Do you use your mobile phone for any financial transactions? | | | YES . . . . . . . . . . . . . . . . . . . . . . . . . . . . . . . . . . . .  NO . . . . . . . . . . . . . . . . . . . . . . . . . . . . . . . . . . . . | 1  2 |  |
| 118 | Do you have an account in a bank or other financial institution that you yourself use? | | | YES . . . . . . . . . . . . . . . . . . . . . . . . . . . . . . . . . . . .  NO . . . . . . . . . . . . . . . . . . . . . . . . . . . . . . . . . . . . | 1  2 |  |
| 119 | Have you ever used the internet? | | | YES . . . . . . . . . . . . . . . . . . . . . . . . . . . . . . . . . . . .  NO . . . . . . . . . . . . . . . . . . . . . . . . . . . . . . . . . . . . | 1  2 | 122 |
| 120 | In the last 12 months, have you used the internet?  IF NECESSARY, PROBE FOR USE FROM ANY LOCATION, WITH ANY DEVICE. | | | YES . . . . . . . . . . . . . . . . . . . . . . . . . . . . . . . . . . . .  NO . . . . . . . . . . . . . . . . . . . . . . . . . . . . . . . . . . . . | 1  2 | 122 |
| 121 | During the last one month, how often did you use the internet: almost every day, at least once a week, less than once a week, or not at all? | | | ALMOST EVERY DAY . . . . . . . . . . . . . . . . . . . . . .  AT LEAST ONCE A WEEK . . . . . . . . . . . . . . . . . . .  LESS THAN ONCE A WEEK . . . . . . . . . . . . . . . . NOT AT ALL . . . . . . . . . . . . . . . . . . . . . . . . . . . | 1  2  3  4 |  |

NO.

122

QUESTIONS AND FILTERS

COUNTRY-SPECIFIC QUESTION ON RELIGION, IF APPROPRIATE.

CODING CATEGORIES

SKIP

1. COUNTRY-SPECIFIC QUESTION ON ETHNICITY, IF APPROPRIATE.
2. In the last 12 months, how many times have you been

(4) away from home for one or more nights?

NUMBER OF TIMES . . . . . . . . . . . . .

NONE 00

201

125

(4)

In the last 12 months, have you been away from home for more than one month at a time?

YES 1

NO

. 2

1. Increase the time reported to the respondent if modules are added to the questionnaire.
2. Revise according to the local education system.
3. Each card should have four simple sentences appropriate to the country (e.g., "Parents love their children.", "Farming is hard work.", "The child is reading a book.", "Children work hard at school."). Cards should be prepared for every language in which respondents are likely to be literate.
4. The question may be considered for deletion in countries with a very low HIV prevalence.


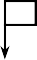

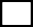

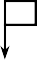

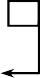


201

206

202

204

203

1. SONS AT HOME . . . . . . . . . . . . . . . .
2. DAUGHTERS AT HOME . . . . . . . .

204

206

205

1. SONS ELSEWHERE . . . . . . . . . .
2. DAUGHTERS ELSEWHERE . . . . .

206

208

207

1. BOYS DEAD . . . . . . . . . . . . . . . .
2. GIRLS DEAD . . . . . . . . . . . . . . . .

TOTAL BIRTHS . . . . . . . . . . . . . . . . . . .

209

226

NO BIRTHS

ONE OR MORE

BIRTHS

CHECK 208:

210

PROBE AND CORRECT 201-208 AS NECESSARY.

NO

YES

CHECK 208:

Just to make sure that I have this right: you have had in TOTAL births during your life. Is that correct?

SUM ANSWERS TO 203, 205, AND 207, AND ENTER TOTAL. IF NONE, RECORD '00'.

208

1. How many boys have died?
2. And how many girls have died?

IF NONE, RECORD '00'.

YES 1

NO 2

Have you ever given birth to a boy or girl who was born alive but later died?

IF NO, PROBE: Any baby who cried, who made any movement, sound, or effort to breathe, or who showed any other signs of life even if for a very short time?

1. How many sons are alive but do not live with you?
2. And how many daughters are alive but do not live with you?

IF NONE, RECORD '00'.

YES 1

NO 2

Do you have any sons or daughters to whom you have given birth who are alive but do not live with you?

1. How many sons live with you?
2. And how many daughters live with you?

IF NONE, RECORD '00'.

YES 1

NO 2

Do you have any sons or daughters to whom you have given birth who are now living with you?

YES 1

NO 2

Now I would like to ask about all the births you have had during your life. Have you ever given birth?

|  |  |
| --- | --- |
|  |  |

|  |  |
| --- | --- |
|  |  |

|  |  |
| --- | --- |
|  |  |

211 Now I would like to record the names of all your births, whether still alive or not, starting with the first one you had.

RECORD NAMES OF ALL THE BIRTHS IN 212. RECORD TWINS AND TRIPLETS ON SEPARATE ROWS. IF THERE ARE MORE THAN 10 BIRTHS, USE AN ADDITIONAL QUESTIONNAIRE, STARTING WITH THE SECOND ROW.

| 212 | 213 | 214 | 215 | 216 | 217  IF ALIVE: | 218  IF ALIVE: | 219  IF ALIVE: | 220  IF DEAD: | 221 |
| --- | --- | --- | --- | --- | --- | --- | --- | --- | --- |
| What | Is | Were | On what day, | Is | How old | Is | RECORD | How old was (NAME) | Were there |
| name was | (NAME) | any of | month, and year | (NAME) | was | (NAME) | HOUSEHOLD | when (he/she) died? | any other |
| given to | a boy or | these | was (NAME) | still | (NAME) at | living | LINE |  | live births |
| your (first/ | a girl? | births | born? | alive? | (NAME)'s | with | NUMBER OF | IF '12 MONTHS' OR | between |
| next) baby?  RECORD NAME.  BIRTH HISTORY NUMBER. |  | twins? |  |  | last birthday?  RECORD AGE IN COMP- LETED YEARS. | you? | CHILD. RECORD '00' IF CHILD NOT LISTED IN HOUSEHOLD. | '1 YR', ASK: Did  (NAME) have (his/her) first birthday?  THEN ASK: Exactly how many months old was (NAME) when (he/she) died?  RECORD DAYS IF LESS THAN 1 MONTH; MONTHS IF LESS THAN TWO  YEARS; OR YEARS. | (NAME OF PREVIOUS  BIRTH) and (NAME),  including any children who died after birth? |

01

BOY 1

GIRL 2

SING 1

MULT 2

DAY MONTH

YEAR

|  |  |  |  |
| --- | --- | --- | --- |

YES 1

NO 2

(SKIP TO 220)

AGE IN YEARS

YES 1

NO 2

HOUSEHOLD LINE NUMBER

(NEXT BIRTH)

DAYS 1

MONTHS 2

YEARS 3

02

BOY 1

GIRL 2

03

BOY 1

GIRL 2

04

BOY 1

GIRL 2

05

BOY 1

GIRL 2

SING 1

MULT 2

SING 1

MULT 2

SING 1

MULT 2

SING 1

MULT 2

DAY MONTH

YEAR DAY

|  |  |  |  |
| --- | --- | --- | --- |

MONTH

|  |  |  |  |
| --- | --- | --- | --- |

YEAR DAY

MONTH

|  |  |  |  |
| --- | --- | --- | --- |

YEAR DAY

MONTH

|  |  |  |  |
| --- | --- | --- | --- |

YEAR

YES 1

NO 2

(SKIP TO 220)

YES 1

NO 2

(SKIP TO 220)

YES 1

NO 2

(SKIP TO 220)

YES 1

NO 2

(SKIP TO 220)

AGE IN YEARS

AGE IN YEARS

AGE IN YEARS

AGE IN YEARS

YES 1

NO 2

YES 1

NO 2

YES 1

NO 2

YES 1

NO 2

HOUSEHOLD LINE NUMBER

(SKIP TO 221)

HOUSEHOLD LINE NUMBER

(SKIP TO 221)

HOUSEHOLD LINE NUMBER

(SKIP TO 221)

HOUSEHOLD LINE NUMBER

(SKIP TO 221)

DAYS 1

MONTHS 2

YEARS 3

DAYS 1

MONTHS 2

YEARS 3

DAYS 1

MONTHS 2

YEARS 3

DAYS 1

MONTHS 2

YEARS 3

YES 1

(ADD BIRTH)

NO 2

(NEXT BIRTH)

YES 1

(ADD BIRTH)

NO 2

(NEXT BIRTH)

YES 1

(ADD BIRTH)

NO 2

(NEXT BIRTH)

YES 1

(ADD BIRTH)

NO 2

(NEXT BIRTH)

| 212 | 213 | 214 | 215 | 216 | 217  IF ALIVE: | 218  IF ALIVE: | 219  IF ALIVE: | 220  IF DEAD: | 221 |
| --- | --- | --- | --- | --- | --- | --- | --- | --- | --- |
| What | Is | Were | On what day, | Is | How old | Is | RECORD | How old was (NAME) | Were there |
| name was | (NAME) | any of | month, and year | (NAME) | was | (NAME) | HOUSEHOLD | when (he/she) died? | any other |
| given to | a boy or | these | was (NAME) | still | (NAME) at | living | LINE |  | live births |
| your (first/ | a girl? | births | born? | alive? | (NAME)'s | with | NUMBER OF | IF '12 MONTHS' OR | between |
| next) baby?  RECORD NAME.  BIRTH HISTORY NUMBER. |  | twins? |  |  | last birthday?  RECORD AGE IN COMP- LETED YEARS. | you? | CHILD. RECORD '00' IF CHILD NOT LISTED IN HOUSEHOLD. | '1 YR', ASK: Did  (NAME) have (his/her) first birthday?  THEN ASK: Exactly how many months old was (NAME) when (he/she) died?  RECORD DAYS IF LESS THAN 1 MONTH; MONTHS IF LESS THAN TWO YEARS; OR YEARS. | (NAME OF PREVIOUS  BIRTH) and (NAME),  including any children who died after birth? |

06

BOY 1

GIRL 2

07

BOY 1

GIRL 2

08

BOY 1

GIRL 2

09

BOY 1

GIRL 2

10

BOY 1

GIRL 2

SING 1

MULT 2

SING 1

MULT 2

SING 1

MULT 2

SING 1

MULT 2

SING 1

MULT 2

DAY MONTH

YEAR DAY

|  |  |  |  |
| --- | --- | --- | --- |

MONTH

|  |  |  |  |
| --- | --- | --- | --- |

YEAR DAY

MONTH

|  |  |  |  |
| --- | --- | --- | --- |

YEAR DAY

MONTH

|  |  |  |  |
| --- | --- | --- | --- |

YEAR DAY

MONTH

|  |  |  |  |
| --- | --- | --- | --- |

YEAR

YES 1

NO 2

(SKIP TO 220)

YES 1

NO 2

(SKIP TO 220)

YES 1

NO 2

(SKIP TO 220)

YES 1

NO 2

(SKIP TO 220)

YES 1

NO 2

(SKIP TO 220)

AGE IN YEARS

AGE IN YEARS

AGE IN YEARS

AGE IN YEARS

AGE IN YEARS

YES 1

NO 2

YES 1

NO 2

YES 1

NO 2

YES 1

NO 2

YES 1

NO 2

HOUSEHOLD LINE NUMBER

(SKIP TO 221)

HOUSEHOLD LINE NUMBER

(SKIP TO 221)

HOUSEHOLD LINE NUMBER

(SKIP TO 221)

HOUSEHOLD LINE NUMBER

(SKIP TO 221)

HOUSEHOLD LINE NUMBER

(SKIP TO 221)

DAYS 1

MONTHS 2

YEARS 3

DAYS 1

MONTHS 2

YEARS 3

DAYS 1

MONTHS 2

YEARS 3

DAYS 1

MONTHS 2

YEARS 3

DAYS 1

MONTHS 2

YEARS 3

YES 1

(ADD BIRTH)

NO 2

(NEXT BIRTH)

YES 1

(ADD BIRTH)

NO 2

(NEXT BIRTH)

YES 1

(ADD BIRTH)

NO 2

(NEXT BIRTH)

YES 1

(ADD BIRTH)

NO 2

(NEXT BIRTH)

YES 1

(ADD BIRTH)

NO 2

(NEXT BIRTH)

| NO. | QUESTIONS AND FILTERS | CODING CATEGORIES | | SKIP |
| --- | --- | --- | --- | --- |
| 222 | Have you had any live births since the birth of (NAME OF LAST BIRTH)? | YES . . . . . . . . . . . . . . . . . . . . . . . . . . . . . . . . . . . .  (RECORD BIRTH(S) IN TABLE) NO . . . . . . . . . . . . . . . . . . . . . . . . . . . . . . . . . . . . | 1 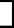 2 |  |
| 223 | COMPARE 208 WITH NUMBER OF BIRTHS IN BIRTH HISTORY  NUMBERS NUMBERS ARE  ARE SAME DIFFERENT 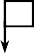 (PROBE AND RECONCILE) 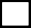 | | |  |
| 224  (1) | CHECK 215: ENTER THE NUMBER OF BIRTHS IN 2010-2015 | NUMBER OF BIRTHS . . . . . . . . . . . . . . . . . . . NONE . . . . . . . . . . . . . . . . . . . . . . . . . . . . . . . . . . . . | 0 | 226 |
| 225  (1) | FOR EACH BIRTH IN 2010-2015, ENTER 'B' IN THE MONTH OF BIRTH IN THE CALENDAR. WRITE  **C** THE NAME OF THE CHILD TO THE LEFT OF THE 'B' CODE. FOR EACH BIRTH, ASK THE NUMBER OF COMPLETED MONTHS THE PREGNANCY LASTED AND RECORD 'P' IN EACH OF THE  PRECEDING MONTHS ACCORDING TO THE DURATION OF PREGNANCY. (NOTE: THE NUMBER OF 'P's MUST BE ONE LESS THAN THE NUMBER OF MONTHS THAT THE PREGNANCY LASTED.) | | |  |
| 226 | Are you pregnant now? | YES . . . . . . . . . . . . . . . . . . . . . . . . . . . . . . . . . . . .  NO . . . . . . . . . . . . . . . . . . . . . . . . . . . . . . . . . . . .  UNSURE . . . . . . . . . . . . . . . . . . . . . . . . . . . . . . | 1  2  8 | 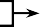 230 |
| 227 | How many months pregnant are you?  RECORD NUMBER OF COMPLETED MONTHS.  **C** ENTER 'P's IN THE CALENDAR, BEGINNING WITH THE MONTH OF  INTERVIEW AND FOR THE TOTAL NUMBER OF COMPLETED MONTHS. | MONTHS . . . . . . . . . . . . . . . . . . . . . . | |  |
| 228 | When you got pregnant, did you want to get pregnant at that time? | YES . . . . . . . . . . . . . . . . . . . . . . . . . . . . . . . . . . . .  NO . . . . . . . . . . . . . . . . . . . . . . . . . . . . . . . . . . . . | 1  2 | 230 |
| 229 | CHECK 208: TOTAL NUMBER OF BIRTHS ONE OR MORE 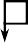 NONE 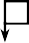  a) Did you want to have a b) Did you want to have a baby later on or did you baby later on or did you not want any more not want any children? children? | LATER . . . . . . . . . . . . . . . . . . . . . . . . . . . . . . . . .  NO MORE/NONE . . . . . . . . . . . . . . . . . . . . . . . . | 1  2 |  |
| 230 | Have you ever had a pregnancy that miscarried, was aborted, or ended in a stillbirth? | YES . . . . . . . . . . . . . . . . . . . . . . . . . . . . . . . . . . . .  NO . . . . . . . . . . . . . . . . . . . . . . . . . . . . . . . . . . . . | 1  2 | 239 |
| 231 | When did the last such pregnancy end? | MONTH . . . . . . . . . . . . . . . . . . . . . . . . YEAR . . . . . . . . . . . . . | |  |

|  |  |  |  |
| --- | --- | --- | --- |

|  |  |  |  |
| --- | --- | --- | --- |

|  |  |  |  |
| --- | --- | --- | --- |

| NO. | QUESTIONS AND FILTERS | CODING CATEGORIES | | SKIP |
| --- | --- | --- | --- | --- |
| 232  (1) | CHECK 231:  LAST PREGNANCY ENDED IN 2010-2015 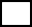 LAST PREGNANCY ENDED IN 2009 OR  EARLIER 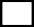 | | | 234  239 |
| LINE NO. | 233  In what month and year did the preceding such pregnancy end? | 234  How many months pregnant were you when that pregnancy ended? | 235 **(1)**  Since January 2010, have you had any other pregnancies that did not result in a live birth? |  |
| 01 |  | NUMBER OF MONTHS | YES 1  NO 2 | NEXT LINE  236 |
| 02 | MONTH YEAR | NUMBER OF MONTHS | YES 1  NO 2 | NEXT LINE  236 |
| 03 | MONTH YEAR | NUMBER OF MONTHS | YES 1  NO 2 | NEXT LINE  236 |
| 04 | MONTH YEAR | NUMBER OF MONTHS | YES 1  NO 2 | 236 |
| 236  (1) | **C** FOR EACH PREGNANCY THAT DID NOT END IN A LIVE BIRTH IN 2010-2015 OR LATER, ENTER 'T' IN THE CALENDAR IN THE MONTH THAT THE PREGNANCY TERMINATED AND 'P' FOR THE  REMAINING NUMBER OF COMPLETED MONTHS OF PREGNANCY.  IF THERE ARE MORE THAN FOUR PREGNANCIES THAT DID NOT END IN A LIVE BIRTH, USE AN ADDITIONAL QUESTIONNAIRE STARTING ON THE SECOND LINE. | | |  |
| 237  (1) | Did you have any miscarriages, abortions or stillbirths that ended before 2010? | YES 1  NO 2 | | 239 |
| 238  (1) | When did the last such pregnancy that terminated before 2010 end? | MONTH . . . . . . . . . . . . . . . . . . . . . . . . YEAR . . . . . . . . . . . . . | |  |

|  |  |
| --- | --- |
|  |  |
|  |  |
|  |  |

| NO. | QUESTIONS AND FILTERS | CODING CATEGORIES | SKIP |
| --- | --- | --- | --- |
| 239 | When did your last menstrual period start?  (DATE, IF GIVEN) | DAYS AGO 1  WEEKS AGO 2  MONTHS AGO 3  YEARS AGO 4  IN MENOPAUSE/  HAS HAD HYSTERECTOMY 994  BEFORE LAST BIRTH 995  NEVER MENSTRUATED 996 |  |
| 240 | From one menstrual period to the next, are there certain days when a woman is more likely to become pregnant? | YES 1  NO 2  DON'T KNOW 8 | 242 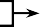 |
| 241 | Is this time just before her period begins, during her period, right after her period has ended, or halfway between two periods? | JUST BEFORE HER PERIOD BEGIN 1  DURING HER PERIOD 2  RIGHT AFTER HER PERIOD HAS ENDE 3  HALFWAY BETWEEN TWO PERIODS 4  OTHER 6  (SPECIFY)  DON'T KNOW 8 |  |
| 242 | After the birth of a child, can a woman become pregnant before her menstrual period has returned? | YES 1  NO 2  DON'T KNOW 8 |  |

1. Year of fieldwork is assumed to be 2015. For fieldwork beginning in 2016, all references to calendar years should be increased by one; for example, 2009 should be changed to 2010, 2010 should be changed to 2011, 2011 should be changed to 2012, and similarly for all years throughout the questionnaire.
