# Supplementary material for: Pregnancy Intentions and Maternal Health Behaviours: Observational Study in 18 African Countries
Source: BJOG. 2025 Sep 10;132(13):2246–55. doi: 10.1111/1471-0528.18367 (PMC12592751; doi:10.1111/1471-0528.18367)
Supplement: Supplementary file 2 — Figure S2: Pregnancy intentions and early ANC. [file BJO-132-2246-s003.docx]

Study

Early ANC in unintended pregnancies

Early ANC in intended pregnancies

Unadjusted odds ratio

Sample size

Adjusted Odds ratio (95% CI) Early ANC

% Weight, IV

1. Burkina Faso
2. Cameroon
3. Cote d'Ivoire
4. Gabon
5. Gambia
6. Ghana
7. Guinea
8. Kenya
9. Liberia
10. Madagascar
11. Mali
12. Mauritania
13. Nigeria
14. Rwanda
15. Senegal
16. Sierra Leone
17. Tanzania
18. Zambia Overall, IV Overall, DL

42.2 (36.0-48.4)

34.9 (29.6-40.2)

32.9 (27.2-38.7)

56.2 (50.5-62.0)

32.0 (26.3-37.6)

50.8 (46.3-55.3)

24.4 (18.7-30.1)

23.6 (20.8-26.5)

63.3 (56.5-70.0)

20.2 (15.3-25.1)

25.7 (20.3-31.2)

47.7 (42.2-53.1)

16.1 (13.0-19.3)

44.7 (40.5-49.0)

48.4 (40.6-56.3)

42.8 (36.7-49.0)

26.1 (22.1-30.0)

33.7 (29.4-38.0)

52.5 (49.6-55.4)

39.3 (35.3-43.3)

38.2 (34.6-41.7)

73.8 (67.8-79.7)

44.4 (40.5-48.3)

67.4 (64.0-70.8)

27.5 (24.2-30.9)

30.9 (27.8-34.1)

75.4 (71.6-79.3)

29.6 (27.1-32.0)

39.0 (35.9-42.1)

59.8 (56.6-63.1)

17.8 (16.3-19.3)

65.1 (61.6-68.5)

62.3 (57.1-67.5)

44.6 (41.5-47.7)

36.0 (32.6-39.4)

38.6 (34.3-42.8)

0.66 (0.51-0.86)

0.83 (0.64-1.07)

0.79 (0.60-1.06)

0.46 (0.31-0.67)

0.59 (0.44-0.79)

0.50 (0.39-0.64)

0.85 (0.62-1.17)

0.69 (0.56-0.85)

0.56 (0.39-0.80)

0.60 (0.44-0.84)

0.54 (0.41-0.72)

0.61 (0.48-0.78)

0.89 (0.71-1.12)

0.43 (0.35-0.54)

0.57 (0.38-0.86)

0.93 (0.71-1.22)

0.63 (0.49-0.81)

0.81 (0.65-1.01)

2366

1902

2149

1285

1798

1891

1534

3958

1130

2532

1933

2312

6280

1546

1255

1931

2174

1960

0.65 (0.49, 0.86)

0.61 (0.47, 0.81)

0.88 (0.65, 1.19)

0.54 (0.36, 0.80)

0.64 (0.47, 0.88)

0.53 (0.40, 0.69)

0.90 (0.64, 1.26)

0.79 (0.64, 0.99)

0.62 (0.42, 0.91)

0.63 (0.45, 0.88)

0.54 (0.40, 0.74)

0.66 (0.52, 0.85)

0.67 (0.52, 0.86)

0.53 (0.41, 0.67)

0.53 (0.32, 0.85)

0.96 (0.72, 1.27)

0.67 (0.51, 0.88)

0.87 (0.69, 1.09)

0.68 (0.63, 0.73)

0.67 (0.62, 0.74)

5.86

6.03

5.03

2.93

4.59

6.40

3.92

9.14

3.06

4.00

4.82

7.24

6.96

7.65

1.96

5.65

6.30

8.46

100.00

(I2 = 44.3%, p = 0.023)


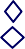

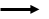

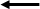


.5 1 1.5

Early ANC less when unintended pregnancy Early ANC more when unintended pregnancy
