# Supplementary material for: Pregnancy Intentions and Maternal Health Behaviours: Observational Study in 18 African Countries
Source: BJOG. 2025 Sep 10;132(13):2246–55. doi: 10.1111/1471-0528.18367 (PMC12592751; doi:10.1111/1471-0528.18367)
Supplement: Supplementary file 4 — Figure S4: Pregnancy intentions and IPTp3+. [file BJO-132-2246-s006.docx]

Study

IPTp3+ in unintended pregnancies

IPTp3+ in intended pregnancies

Unadjusted odds ratio

Sample size

Adjusted Odds ratio (95% CI) IPTp3+

% Weight, IV

1. Burkina Faso
2. Cameroon
3. Cote d'Ivoire
4. Gabon
5. Gambia
6. Ghana
7. Guinea
8. Kenya
9. Liberia
10. Madagascar
11. Mali
12. Mauritania
13. Nigeria
14. Senegal
15. Sierra Leone
16. Tanzania
17. Zambia Overall, IV Overall, DL

54.9 (48.9-61.0)

28.9 (24.3-33.6)

34.8 (28.1-41.5)

34.8 (28.0-41.5)

52.2 (46.1-58.4)

54.0 (48.6-59.5)

30.9 (24.5-37.2)

5.9 (4.5-7.3)

39.7 (34.3-45.1)

21.7 (17.0-26.4)

27.6 (21.8-33.4)

7.3 (4.6-9.9)

19.4 (16.0-22.8)

17.4 (9.5-25.3)

36.3 (29.4-43.3)

28.7 (24.3-33.0)

57.8 (53.4-62.2)

55.6 (52.5-58.6)

30.9 (27.5-34.2)

34.1 (30.3-37.9)

37.5 (31.0-44.0)

54.6 (51.3-58.0)

68.4 (64.6-72.2)

38.0 (34.5-41.6)

5.7 (4.7-6.7)

39.8 (33.9-45.7)

34.3 (31.4-37.2)

27.7 (24.8-30.6)

11.5 (9.1-14.0)

15.9 (14.5-17.3)

21.5 (17.9-25.1)

32.4 (29.0-35.7)

34.8 (31.1-38.5)

59.8 (56.1-63.4)

0.97 (0.75-1.27)

0.91 (0.70-1.19)

1.03 (0.78-1.37)

0.89 (0.61-1.29)

0.91 (0.68-1.22)

0.54 (0.42-0.70)

0.73 (0.53-1.00)

1.04 (0.75-1.43)

1.00 (0.71-1.39)

0.53 (0.40-0.72)

1.00 (0.74-1.33)

0.60 (0.39-0.93)

1.27 (1.02-1.59)

0.77 (0.44-1.34)

1.19 (0.89-1.59)

0.75 (0.58-0.97)

0.92 (0.74-1.15)

2366

1902

2149

1285

1798

1891

1534

3958

1130

2532

1933

2312

6280

1255

1931

2174

1960

0.97 (0.73, 1.29)

0.80 (0.60, 1.05)

1.09 (0.81, 1.48)

0.94 (0.63, 1.41)

0.93 (0.68, 1.26)

0.59 (0.45, 0.78)

0.67 (0.48, 0.93)

0.95 (0.68, 1.33)

1.03 (0.73, 1.46)

0.54 (0.40, 0.73)

1.02 (0.75, 1.38)

0.59 (0.38, 0.92)

0.97 (0.76, 1.24)

0.84 (0.47, 1.48)

1.20 (0.88, 1.63)

0.80 (0.62, 1.04)

1.02 (0.80, 1.28)

0.87 (0.81, 0.94)

0.86 (0.77, 0.97)

6.93

7.17

5.90

3.37

5.83

7.23

5.06

4.78

4.52

5.95

5.88

2.74

9.03

1.69

5.73

8.20

10.01

100.00

(I^2^ = 54.3%, p = 0.004)


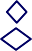

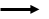

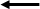


.5 1 1.5

IPTp3+ less when unintended pregnancy IPTp 3+ more when unintended pregnancy
