# Supplementary material for: Pregnancy Intentions and Maternal Health Behaviours: Observational Study in 18 African Countries
Source: BJOG. 2025 Sep 10;132(13):2246–55. doi: 10.1111/1471-0528.18367 (PMC12592751; doi:10.1111/1471-0528.18367)
Supplement: Supplementary file 5 — Figure S5: Pregnancy intentions and receipt of tetanus toxoid. [file BJO-132-2246-s005.docx]

Study

Received tetanus in unintended pregnancies

Received tetanus in intended pregnancies

Unadjusted odds ratio

Sample size

Adjusted Odds ratio (95% CI) Tetanus

% Weight, IV

1. Burkina Faso
2. Cameroon
3. Cote d'Ivoire
4. Gabon
5. Gambia
6. Ghana
7. Guinea
8. Kenya
9. Liberia
10. Madagascar
11. Mali
12. Mauritania
13. Nigeria
14. Rwanda
15. Senegal
16. Sierra Leone
17. Tanzania
18. Zambia Overall, IV Overall, DL

82.7 (77.9-87.4)

77.7 (73.3-82.1)

84.8 (81.2-88.4)

79.2 (74.3-84.1)

77.3 (72.3-82.3)

82.9 (78.9-87.0)

69.2 (62.7-75.6)

87.5 (85.6-89.5)

89.4 (85.8-93.0)

61.0 (54.7-67.3)

66.2 (60.3-72.1)

53.4 (47.5-59.3)

76.6 (73.3-79.8)

65.6 (61.4-69.9)

88.7 (83.9-93.5)

97.3 (95.3-99.3)

74.0 (70.0-77.9)

75.6 (71.9-79.2)

85.0 (83.0-86.9)

72.6 (69.2-76.0)

84.6 (82.0-87.2)

82.5 (77.2-87.7)

85.4 (82.8-88.0)

88.5 (86.0-90.9)

80.8 (77.4-84.2)

88.5 (86.9-90.2)

93.8 (91.5-96.0)

67.8 (65.3-70.3)

77.2 (74.0-80.3)

62.7 (59.1-66.3)

70.5 (68.4-72.6)

77.3 (74.3-80.3)

87.7 (84.9-90.6)

97.9 (97.0-98.8)

81.3 (78.6-84.0)

75.3 (71.7-78.9)

0.84 (0.59-1.21)

1.32 (0.99-1.75)

1.01 (0.73-1.40)

0.81 (0.51-1.29)

0.58 (0.40-0.84)

0.63 (0.45-0.89)

0.53 (0.39-0.73)

0.91 (0.71-1.16)

0.56 (0.32-0.97)

0.74 (0.56-0.98)

0.58 (0.44-0.77)

0.68 (0.54-0.87)

1.37 (1.12-1.67)

0.56 (0.44-0.72)

1.10 (0.62-1.93)

0.76 (0.31-1.84)

0.65 (0.50-0.85)

1.01 (0.77-1.33)

2364

1902

2146

1285

1798

1891

1534

3956

1130

2532

1933

2312

6280

1546

1255

1931

2173

1960

0.78 (0.53, 1.15)

0.99 (0.72, 1.38)

0.96 (0.66, 1.38)

0.79 (0.46, 1.34)

0.63 (0.42, 0.96)

0.58 (0.40, 0.83)

0.49 (0.35, 0.71)

0.87 (0.66, 1.15)

0.53 (0.27, 1.03)

0.72 (0.54, 0.97)

0.53 (0.38, 0.74)

0.73 (0.57, 0.95)

0.76 (0.60, 0.95)

0.66 (0.50, 0.87)

1.16 (0.64, 2.12)

0.71 (0.26, 1.90)

0.66 (0.50, 0.87)

1.02 (0.78, 1.34)

0.74 (0.68, 0.80)

0.74 (0.66, 0.82)

4.23

5.84

4.56

2.17

3.67

4.71

4.92

8.01

1.40

6.94

5.87

9.57

11.70

7.78

1.72

0.64

7.92

8.34

100.00

(I^2^ = 38.5%, p = 0.050)


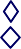

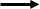

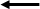


.5 1 1.5

Received tetanus less when unintended pregnancy Received tetanus more when unintended pregnancy
