# Supplementary material for: Pregnancy Intentions and Maternal Health Behaviours: Observational Study in 18 African Countries
Source: BJOG. 2025 Sep 10;132(13):2246–55. doi: 10.1111/1471-0528.18367 (PMC12592751; doi:10.1111/1471-0528.18367)
Supplement: Supplementary file 6 — Figure S6: Pregnancy intentions and mosquito net use during pregnancy. [file BJO-132-2246-s002.docx]

Study

Net use

in unintended pregnancies

Net use

in intended pregnancies

Unadjusted odds ratio

Sample size

Adjusted Odds ratio (95% CI) Net use

% Weight, IV

1. Burkina Faso
2. Cameroon
3. Cote d'Ivoire
4. Gabon
5. Gambia
6. Ghana
7. Guinea
8. Kenya
9. Liberia
10. Madagascar
11. Mali
12. Mauritania
13. Nigeria
14. Rwanda
15. Senegal
16. Sierra Leone
17. Tanzania
18. Zambia Overall, IV Overall, DL

88.3 (81.1-95.5)

76.2 (68.7-83.7)

No information

65.8 (55.3-76.4)

54.1 (43.5-64.8)

77.3 (70.1-84.6)

57.7 (38.3-77.1)

No information

77.6 (65.7-89.4)

76.6 (64.4-88.8)

92.4 (87.7-97.2)

38.4 (27.1-49.6)

76.4 (70.9-81.9)

85.9 (78.7-93.0)

91.3 (85.0-97.5)

85.3 (73.2-97.4)

84.0 (77.7-90.3)

67.9 (60.0-75.7)

85.6 (80.5-90.8)

80.6 (75.8-85.4)

68.6 (53.0-84.2)

60.9 (52.4-69.4)

66.2 (58.8-73.7)

62.5 (53.1-71.9)

80.0 (67.4-92.6)

85.6 (80.4-90.9)

92.3 (89.5-95.2)

29.4 (20.5-38.3)

84.8 (82.8-86.7)

94.9 (87.9-101.9)

82.5 (76.3-88.8)

94.6 (90.7-98.5)

90.6 (85.6-95.7)

74.1 (66.9-81.3)

1.27 (0.56-2.85)

0.77 (0.46-1.28)

0.88 (0.38-2.07)

0.76 (0.43-1.32)

1.74 (1.02-2.96)

0.82 (0.34-1.96)

0.86 (0.31-2.41)

0.55 (0.25-1.22)

1.02 (0.46-2.23)

1.49 (0.79-2.82)

0.58 (0.41-0.81)

0.33 (0.07-1.53)

2.21 (0.91-5.37)

0.33 (0.10-1.11)

0.54 (0.25-1.15)

0.74 (0.44-1.24)

258

389

114

213

285

130

89

221

458

175

1534

131

223

164

259

280

1.35 (0.56, 3.26)

0.97 (0.56, 1.67)

0.68 (0.22, 2.12)

0.79 (0.41, 1.52)

1.88 (0.99, 3.56)

1.17 (0.42, 3.25)

1.01 (0.24, 4.24)

0.58 (0.24, 1.40)

1.25 (0.53, 2.93)

1.59 (0.78, 3.26)

0.84 (0.58, 1.21)

0.31 (0.03, 2.92)

1.98 (0.72, 5.42)

0.37 (0.07, 2.06)

0.57 (0.26, 1.27)

0.90 (0.49, 1.63)

0.97 (0.81, 1.17)

0.98 (0.81, 1.18)

4.17

10.83

1.77

2.53

7.68

7.92

3.09

1.77

1.57

4.12

4.47

6.31

24.72

0.64

3.19

1.09

5.11

9.01

100.00

(I^2^ = 0.0%, p = 0.416)


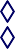

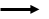

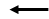


.5 1 1.5

Used net less when unintended pregnancy Used net more when unintended pregnancy
