# Supplementary material for: Pregnancy Intentions and Maternal Health Behaviours: Observational Study in 18 African Countries
Source: BJOG. 2025 Sep 10;132(13):2246–55. doi: 10.1111/1471-0528.18367 (PMC12592751; doi:10.1111/1471-0528.18367)
Supplement: Supplementary file 7 — Figure S7: Pregnancy intentions and immediate breastfeeding. [file BJO-132-2246-s007.docx]

Immediate breastfeeding in unintended

Immediate breastfeeding in

Unadjusted

Sample

Adjusted Odds ratio

% Weight,

Study

pregnancies

intended pregnancies

odds ratio

size

(95% CI) Breastfeeding IV

1. Burkina Faso
2. Cameroon
3. Cote d'Ivoire
4. Gabon
5. Gambia
6. Ghana
7. Guinea
8. Kenya
9. Liberia
10. Madagascar
11. Mali
12. Mauritania
13. Nigeria
14. Rwanda
15. Senegal
16. Sierra Leone
17. Tanzania
18. Zambia Overall, IV Overall, DL

61.6 (56.2-67.1)

44.9 (40.3-49.4)

43.4 (39.3-47.6)

73.9 (70.5-77.3)

35.9 (31.0-40.9)

50.2 (46.4-54.1)

27.7 (22.1-33.2)

66.6 (63.0-70.1)

68.1 (64.1-72.1)

53.7 (48.4-59.0)

57.7 (52.3-63.2)

53.5 (49.2-57.8)

44.3 (40.8-47.7)

86.7 (84.1-89.4)

18.3 (12.5-24.0)

71.7 (66.9-76.4)

67.6 (63.9-71.2)

74.7 (71.7-77.7)

62.2 (60.1-64.4)

51.6 (49.0-54.3)

46.6 (44.2-49.1)

78.5 (75.1-81.9)

40.1 (37.5-42.6)

52.0 (49.2-54.8)

39.0 (36.2-41.7)

64.7 (62.2-67.2)

66.9 (63.2-70.7)

60.8 (58.8-62.9)

68.8 (66.5-71.1)

61.6 (59.3-63.9)

42.4 (41.1-43.8)

86.0 (83.7-88.3)

26.7 (24.0-29.3)

79.6 (77.6-81.6)

66.8 (64.4-69.2)

80.1 (77.8-82.5)

0.94 (0.80-1.12)

0.73 (0.63-0.84)

0.96 (0.84-1.10)

0.68 (0.57-0.82)

0.78 (0.66-0.92)

0.96 (0.85-1.09)

0.55 (0.45-0.68)

1.05 (0.92-1.20)

1.03 (0.87-1.21)

0.80 (0.68-0.94)

0.69 (0.59-0.82)

0.72 (0.63-0.82)

1.17 (1.07-1.29)

1.33 (1.09-1.61)

0.56 (0.43-0.74)

0.58 (0.49-0.69)

0.97 (0.84-1.10)

0.71 (0.62-0.81)

5409

4293

4631

2728

3705

4646

3403

4378

2660

5583

4683

4847

16345

3698

3031

4759

4681

5090

0.93 (0.78, 1.11)

0.73 (0.63, 0.85)

1.02 (0.89, 1.18)

0.68 (0.56, 0.82)

0.81 (0.68, 0.96)

0.97 (0.85, 1.10)

0.52 (0.42, 0.64)

1.10 (0.95, 1.27)

1.10 (0.93, 1.31)

0.79 (0.67, 0.93)

0.67 (0.56, 0.79)

0.68 (0.59, 0.78)

0.93 (0.84, 1.03)

1.22 (0.99, 1.51)

0.53 (0.40, 0.71)

0.62 (0.52, 0.74)

0.96 (0.83, 1.10)

0.66 (0.57, 0.76)

0.83 (0.80, 0.87)

0.81 (0.73, 0.90)

4.27

6.29

6.59

3.76

4.47

8.13

3.00

6.57

4.55

5.01

4.76

6.94

13.37

3.00

1.71

4.16

6.72

6.71

100.00

(I^2^ = 87.5%, p < 0.001)


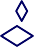

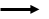

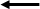


.5 1 1.5

Immediate breastfeeding (bf) less when unintended|Immediate bf more when unintended pregnancy
